# Association between the number of convenience stores and caries status in permanent teeth among elementary school children: results from the A-CHILD population-based longitudinal cohort study

**DOI:** 10.3389/fpubh.2023.1228197

**Published:** 2023-10-26

**Authors:** Nobutoshi Nawa, Hisaaki Nishimura, Yusuke Matsuyama, Satomi Doi, Aya Isumi, Takeo Fujiwara

**Affiliations:** ^1^Department of Global Health Promotion, Tokyo Medical and Dental University (TMDU), Tokyo, Japan; ^2^Department of Health Policy, Tokyo Medical and Dental University (TMDU), Tokyo, Japan

**Keywords:** caries, geographic information systems, Japan, social epidemiology, longitudinal study

## Abstract

**Background:**

In Japan, people have rich access to 24-h convenience stores where they can buy sweets, juice and fluoride hygiene products, among others. However, the association between the number of convenience stores in a neighbourhood and caries in elementary school children status has not been studied. This study aimed to investigate this particular association.

**Methods:**

Data were derived from a population-based longitudinal cohort study (A-CHILD study) of elementary school children from first-grade to fourth-grade in Adachi City, Tokyo. Caregivers were asked to complete a questionnaire in 2015, 2016, and 2018. A total of 3,136 caregivers provided a valid response. We analysed the association using multilevel Poisson regression.

**Results:**

The mean number of caries among children in school districts with low, middle, and high number of convenience stores was 0.31 (SD: 0.81), 0.21 (SD: 0.69), and 0.16 (SD: 0.58). After covariate adjustment, children in the school districts with high and middle number of convenience stores had 44% (mean ratio 0.56, 95% CI: 0.31, 0.998) and 31% (mean ratio 0.69, 95% CI: 0.42, 1.13) fewer caries in their permanent teeth, respectively, than children in the school districts with low number of convenience stores. We also found dose–response relationship (*p* for trend: 0.042).

**Conclusion:**

Higher number of convenience stores in a school district was associated with fewer caries in permanent teeth among elementary school children. Further study elucidating the mechanism on this protective association is warranted.

## Introduction

Dental caries is the most common disease in the world, with a prevalence of about 50% in permanent teeth of children worldwide (1–3). In Japan, the prevalence of dental caries was reported to be up to 40% among elementary school children (4). The cost of treating dental diseases under Japan’s universal health insurance system in 2019 was approximately 27 billion USD (5, 6). Dental caries is a major health burden for children as it can lead to malnutrition and poor quality of life (7). Poor oral health has also been linked to school absenteeism and poor academic performance in children (8). Therefore, research to elucidate the risk factors for dental caries in children is important.

Many prior studies have focused on the risk factors for dental caries at the individual level (1, 9); relatively fewer studies have examined the risk factors at the community level. Among them, a decent number of studies have examined the association between area-level deprivation and dental caries (10–12). Further, a prior study that examined area-level risk factors has also looked at the association of school-based fluoride intervention programs (13) with dental caries in children. Interestingly, one study used questionnaires to ask individuals about their vending machine use and reported that vending machine use increased the risk of dental caries (14). However, since people nowadays shop at convenience stores rather than through vending machines, it is better to consider their access to convenience stores (15). Notably, the association between the number of convenience stores in a neighbourhood and the caries status has not been clarified. While the use of self-reported questionnaires to assess the local food environment can be reliable if administered properly, it is more desirable to use objective information using geographic information systems (GIS) (16).

In Japan, people have rich access to convenience stores. There are approximately 56,000 convenience stores in Japan, one store for every 2,246 people or a ratio of 0.45 stores for every 1,000 people (17). Unlike convenience stores in the US, which are often attached to gas stations, Japanese convenience stores are independent and most of them open 24 h a day. The high quality of products sold, especially food, was reported by several American media outlets during the 2020 Tokyo Olympics, including CNN and The New York Times (18, 19). In fact, at convenience stores across Japan, people can purchase a variety of food, fruits, snacks, juices, and even fluoridated hygiene products. Further, GIS information of convenience stores is also available in the Japanese Yellow Pages database (NTT Town Page database by NTT Town Page Inc. in Tokyo) (20, 21). Therefore, we have set up an empirical study in Japan to investigate the association between the number of convenience stores and dental caries status. The study objective was to investigate the association between the number of convenience stores in a neighbourhood and the caries status among elementary school children in Japan.

## Methods

### Sample

**Figure 1:**
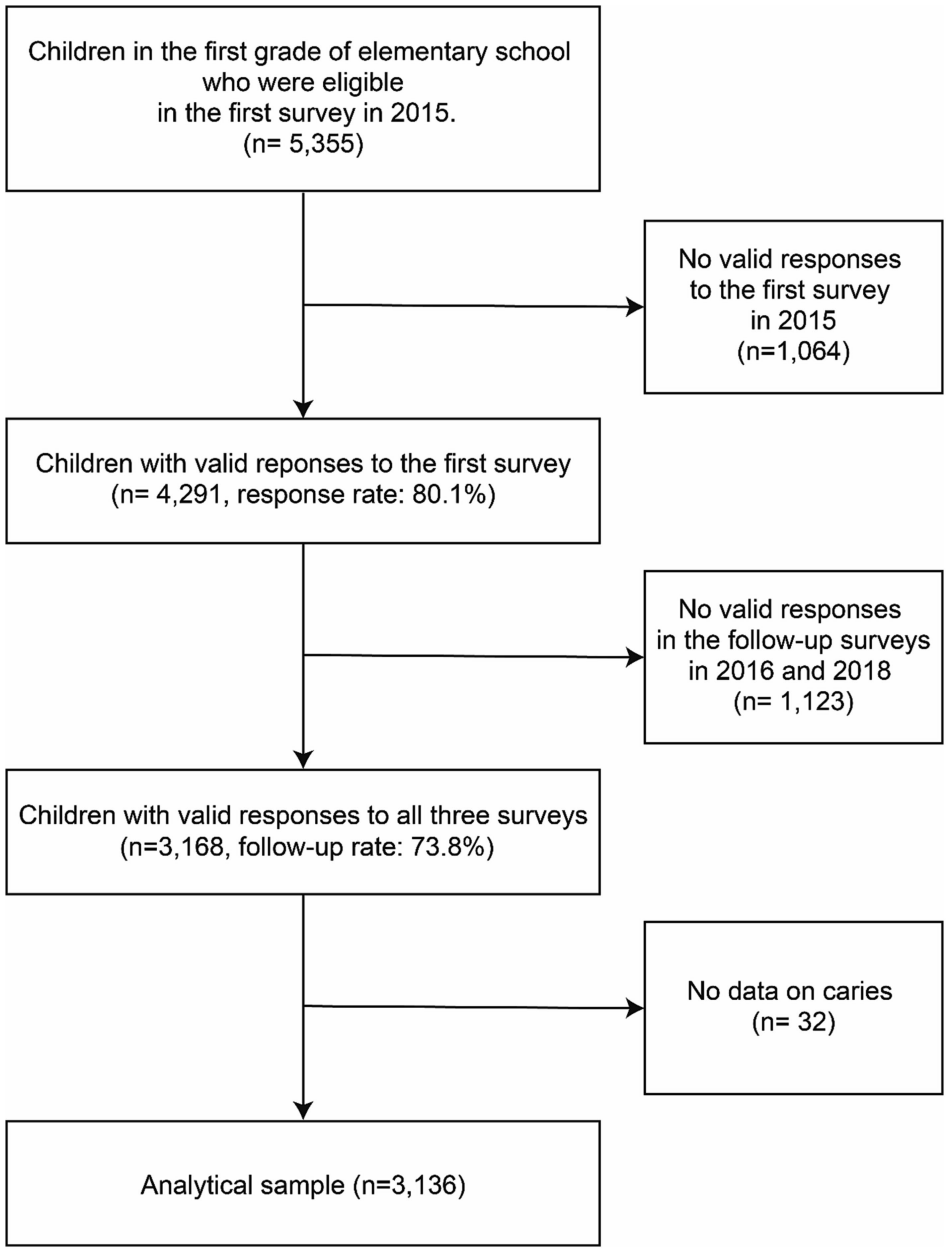
Flowchart of participants. We analysed data from 3,136 elementary school children in Adachi City who provided valid responses to questionnaires administered in 2015, 2016, and 2018 and to whom we were able to link information about their caries status.

Data were derived from the Adachi Child Health Impact of Living Difficulty (A-CHILD) study, a population-based longitudinal cohort study of elementary school children in Adachi City, Tokyo (22). Details of how the longitudinal cohort study was conducted were reported in the cohort paper (22). In short, the longitudinal cohort study began in 2015 with a complete-sample survey of first-grade children (6–7 years old) in all public elementary schools in Adachi City, Tokyo, Japan. Questionnaires were completed by the caregivers of all eligible children. Children who completed the survey returned it in anonymous sealed envelopes at their schools. After the 2015 survey, a follow-up survey was conducted with children who participated in the first wave. Assuming an average number of caries (from 0.5 to 0.37) for children in school districts with the lowest number of convenience stores, a mean ratio of 0.2–0.4, a significance level of 0.05, statistical power of at least 80%, after multiplying by 1.5 to account for intermediate groups (the exposure variable of interest has three categories), and a margin of 10%, the sample size was estimated to be sufficient for 3,129 or more. As shown in Figure 1, children in the first grade of all 69 elementary schools in Adachi City in 2015 were eligible for the first questionnaire survey (*n* = 5,355). We obtained 4,291 valid responses from caregivers for the first questionnaire survey (response rate: 80.1%). Caregivers were then asked to complete a questionnaire in 2016 and 2018, and 3,168 of them provided a valid response in all 3 years (follow-up rate: 73.8%). This survey data was also linked to information on children’s health status, such as dental caries, obtained through annual school checkups. For those who responded to the survey and agreed to participate in the study, the exclusion criterion for this study was the presence of missing values in the outcome variable. We excluded 32 responses due to missing on caries data in 2015 and 2018, resulting in an analytic sample size of 3,136. This study was approved by the Ethics Committee at the National Center for Child Health and Development (Study ID: 1147) and Tokyo Medical and Dental University (Study ID: M2016-284).

## Measures

### Exposure variable

#### Number of convenience stores in a school district

Information on the address of convenience stores in 2018 was derived from the Japanese Yellow Pages database of phone numbers, addresses, and job titles (NTT TownPage database by NTT TownPage Corporation, Tokyo, Japan) (20, 21). Each address was geocoded based on ArcGIS Online World Geocoding Service (ESRI, Redlands, CA) and Google Earth (Google, Mountain View, CA). Geographic data on school district boundaries in 2016 was obtained from National Land Numerical Information by the Ministry of Land Infrastructure Transport and Tourism of Japan (23). The number of convenience stores in a school district was counted using ArcGIS Pro version 2.6 (ESRI Japan, Tokyo, Japan).

### Outcome variable

#### The number of dental caries in 2018

In Japan, dental checkups by school dentists are included in the mandatory annual school health checkups. Dental checkups must follow national guidelines (24), and checkups are conducted using dental mirrors and ball-end probes or community periodontal index probes. In this study, data on the number of dental caries (i.e., tooth decay or filled teeth) of permanent teeth in children in 2018 was used as the outcome. The information was extracted from the linked dental checkup data.

### Covariates

Covariates included maternal age, education, paternal education, household income, marital status, parental psychological distress, child sex, birth order, baseline number of caries in permanent teeth (in 2015) (25, 26) and number of dental clinics in 2014 and land prices in a school district in 2016. Information on maternal age (<30, 30–35, 35–40, ≥45, missing), maternal education (high school or less, some college, college or more, others/unknown/missing), paternal education (high school or less, some college, college or more, others/unknown/missing), household income (<3, 3–6, 6–10, ≥10 million JPY, unknown/missing), marital status (married, single/divorced/widowed/other, missing), parental psychological distress, child sex, birth order (only child, first-born, middle-born, last-born, missing) was obtained by the questionnaire, while information on baseline number of caries in permanent teeth (in 2015) was extracted from the linked dental checkup data.

Geographical data on dental clinic addresses, school district boundaries, and land prices were derived from National Land Numerical Information by Ministry of Land Infrastructure Transport and Tourism of Japan (23). Number of dental clinics in a school district was counted and land prices in a school district was calculated using ArcGIS Pro version 2.8 (ESRI Japan, Tokyo, Japan). These variables were used as categorical variables, except for baseline number of caries in permanent teeth (in 2015) and land prices in a school district, which were used as continuous variables (Table 1).

**Table 1:** Demographic characteristics of study participants by the number of convenience stores in a school district (*n* = 3,136).

		Children in school districts with a low number of convenience stores (the lowest tertile: 0–2 stores; *n* = 1,080)	Children in school districts with a middle number of convenience stores (the middle tertile: 3–4 stores; *n* = 1,205)	Children in school districts with a high number of convenience stores (the highest tertile: 5–15 stores; *n* = 851)	*p*-value^a^
Variable		*N* (%) or Mean (SD)	*N* (%) or Mean (SD)	*N* (%) or Mean (SD)	
Maternal age (years)	<30	47 (4.4)	54 (4.5)	36 (4.2)	0.188
	30–35	180 (16.7)	202 (16.8)	131 (15.4)	
	35–40	397 (36.8)	417 (34.6)	293 (34.4)	
	40–45	337 (31.2)	387 (32.1)	276 (32.4)	
	≥45	107 (9.9)	109 (9.1)	95 (11.2)	
	Missing	12 (1.1)	36 (3.0)	20 (2.4)	
Maternal education	High school or less	362 (33.5)	432 (35.9)	277 (32.6)	**<0.001**
	Some college	484 (44.8)	508 (42.2)	332 (39.0)	
	College or more	214 (19.8)	224 (18.6)	226 (26.6)	
	Others/unknown/missing	20 (1.9)	41 (3.4)	16 (1.9)	
Paternal education	High school or less	395 (36.6)	441 (36.6)	297 (34.9)	0.072
	Some college	208 (19.3)	227 (18.8)	133 (15.6)	
	College or more	415 (38.4)	444 (36.9)	357 (42.0)	
	Others/unknown/missing	62 (5.7)	93 (7.7)	64 (7.5)	
Annual household income (JPY)	<3 million	104 (9.6)	120 (10.0)	88 (10.3)	0.412
	3–6 million	453 (41.9)	493 (40.9)	330 (38.8)	
	6–10 million	346 (32.0)	367 (30.5)	266 (31.3)	
	≥10 million	91 (8.4)	100 (8.3)	88 (10.3)	
	Unknown/missing	86 (8.0)	125 (10.4)	79 (9.3)	
Marital status	Married	991 (91.8)	1,088 (90.3)	770 (90.5)	0.075
	Single/divorced/widowed/other	58 (5.4)	89 (7.4)	67 (7.9)	
	Missing	31 (2.9)	28 (2.3)	14 (1.7)	
Parental psychological distress	K6 5+	298 (27.6)	322 (26.7)	246 (28.9)	0.585
	K6 < 5	773 (71.6)	868 (72.0)	599 (70.4)	
	Missing	9 (0.8)	15 (1.2)	6 (0.7)	
Child sex	Male	555 (51.4)	611 (50.7)	413 (48.5)	0.438
	Female	525 (48.6)	594 (49.3)	438 (51.5)	
Birth order	Only child (no siblings)	591 (54.7)	618 (51.3)	458 (53.8)	0.284
	First-born (having only younger siblings)	77 (7.1)	82 (6.8)	43 (5.1)	
	Middle-born (having both older and younger siblings)	98 (9.1)	132 (11.0)	93 (10.9)	
	Last-born (having only older siblings)	107 (9.9)	110 (9.1)	79 (9.3)	
	Missing	207 (19.2)	263 (21.8)	178 (20.9)	
Number of dental clinics in a school district	The lowest tertile (0–3 clinics)	570 (52.8)	325 (27.0)	92 (10.8)	**<0.001**
	The middle tertile (4–6 clinics)	360 (33.3)	517 (42.9)	313 (36.8)	
	The highest tertile (7–27 clinics)	150 (13.9)	363 (30.1)	446 (52.4)	
Land prices in a school district (JPY/m^2^)	Mean (SD)	288,773 (55,598)	278,288 (65,156)	399,491 (196,431)	**<0.001**
Number of caries in permanent teeh in 2018	Mean (SD)	0.31 (0.81)	0.21 (0.69)	0.16 (0.58)	**<0.001**
Number of caries in permanent teeh in 2015	Mean (SD)	0.04 (0.25)	0.05 (0.31)	0.03 (0.23)	0.161

a To compare values across different groups, a chi-square test was used for categorical variables and ANOVA for continuous variables. Bold indicates *p* < 0.05.

Parental psychological distress was assessed with the Kessler 6 (K6), a six-item scale for screening psychological distress; total K6 scores ranges from 0 to 24, with higher scores indicating greater psychological distress (27, 28). In the present study, the Japanese version of the K6 was used (27, 28).

### Analysis

First, to compare values across different groups, a chi-square test was used for categorical variables and ANOVA for continuous variables. Then, a multilevel Poisson regression model with a random intercept was used to estimate the association between the number of convenience stores and the number of caries in permanent teeth among elementary school children in Japan. The following covariates were included in the adjusted models: maternal age, education, paternal education, household income, marital status, parental psychological distress, child sex, birth order, baseline number of caries in permanent teeth (in 2015) and number of dental clinics and land prices in a school district. Dummy variables for missing values were created for these covariates, except for the baseline number of caries in permanent teeth (in 2015).

Further, while convenience stores pose the advantages for many people due to easy geographical access with most being open 24 h a day, products are usually sold at a fixed price without discount, except for discount for some food products that are approaching their expiry date (29). Therefore, considering the possibility that people from low-income households may tend to avoid buying certain products at neighborhood convenience stores, even if the convenience stores are close by (30, 31), additional analysis was conducted to stratify the analysis according to household income. Low household income was defined as household income below JPY3 million (about USD27,000) per year, based on 50% of the median national household income (32). All analyses were conducted using STATA 17 (StataCorp LP, College Station, TX, United States).

## Results

Table 1 shows demographic characteristics of study participants by the number of convenience stores in a school district (*n* = 3,136). Although the age of the mothers was similar among the three groups, the group with the highest number of convenience stores in the school district had the highest percentage of mothers with college or more education (19.8%, 18.6%, and 26.6% in the groups with the lowest, medium, and highest number of convenience stores, respectively). There were no differences in paternal education, household income, marital status, parental psychological distress, child gender, or birth order among the three groups. The group with the highest number of convenience stores in the school district had the highest percentage of dental clinics in the school district (13.9% in the group with the lowest number of convenience stores, 30.1% in the medium group, and 52.4% in the highest group) and the highest land prices. The group with the lowest number of convenience stores had an average land price of 288,773 (SD: 55,598) JPY/m^2^, the medium group 278,288 (SD: 65,156) JPY, and the highest group 399,491 (SD: 196,431) JPY. The mean number of dental caries in the permanent teeth for children in school districts with the fewest, middle, and highest number of convenience stores was 0.31 (SD: 0.81), 0.21 (SD: 0.69), and 0.16 (SD: 0.58).

Table 2 shows the association between the number of convenience stores in a school district and the number of caries in permanent teeth among fourth-grade elementary school children. Before covariate adjustment, children in the school districts with the highest and middle number of convenience stores had 45% (mean ratio 0.55, 95% CI: 0.31, 0.98) and 28% (mean ratio 0.72, 95% CI: 0.42, 1.22) fewer caries in their permanent teeth, respectively, than children in the school districts with the lowest number of convenience stores. After covariate adjustment, children in the school districts with the highest and middle number of convenience stores had 44% (mean ratio 0.56, 95% CI: 0.31, 0.998) and 31% (mean ratio 0.69, 95% CI: 0.42, 1.13) fewer caries in their permanent teeth, respectively, than children in the school districts with the lowest number of convenience stores. The *p* for trend was 0.042, suggesting that there was a dose–response relationship.

**Table 2:** Association between the number of convenience stores in a school district and the number of caries in permanent teeth among fourth-grade elementary school children (*n* = 3,136) in 2018.

	Crude		Adjusted^a^	
	Mean ratio	95%CI	Mean ratio	95%CI
School districts with the low number of convenience stores (the lowest tertile: 0–2 stores)	Ref		Ref	
School districts with the middle number of convenience stores (the middle tertile: 3–4 stores)	0.72	0.42, 1.22	0.69	0.42, 1.13
School districts with the high number of convenience stores (the highest tertile: 5–15 stores)	**0.55**	**0.31, 0.98**	**0.56**	**0.31, 0.998**
*p* for trend	**0.040**		**0.042**	
Maternal age (in years):				
<30	Ref		Ref	
30–35	1.16	0.80, 1.70	1.37	0.92, 2.02
35–40	0.82	0.57, 1.18	1.03	0.70, 1.52
40–45	1.13	0.79, 1.63	1.39	0.94, 2.05
≥45	0.90	0.59, 1.38	1.13	0.72, 1.78
Maternal education:				
High school or less	Ref		Ref	
Some college	**0.80**	**0.68, 0.94**	**0.83**	**0.70, 0.99**
College or more	**0.71**	**0.57, 0.89**	**0.77**	**0.60, 0.98**
Others/unknown/missing	1.01	0.65, 1.56	0.99	0.60, 1.62
Paternal education:				
High school or less	Ref		Ref	
Some college	0.85	0.69, 1.05	0.85	0.68, 1.05
College or more	0.84	0.71, 1.01	0.91	0.74, 1.11
Others/unknown/missing	1.15	0.88, 1.51	0.99	0.65, 1.52
Annual household income (JPY):				
<3 million	Ref		Ref	
3–6 million	0.90	0.71, 1.14	1.06	0.80, 1.40
6–10 million	**0.70**	**0.55, 0.91**	0.83	0.61, 1.13
≥10 million	0.82	0.59, 1.14	0.99	0.68, 1.45
Unknown/missing	0.90	0.66, 1.23	0.94	0.67, 1.33
Marital status:				
Single/divorced/widowed/other	Ref		Ref	
Married	**0.73**	**0.56, 0.93**	0.73	0.47, 1.12
Parental psychological distress:				
K6 5+	Ref		Ref	
K6 < 5	0.88	0.75, 1.03	0.94	0.80, 1.11
Child sex:				
Male	Ref		Ref	
Female	**1.18**	**1.02, 1.37**	1.15	0.99, 1.34
Birth order:				
Only child (no siblings)	Ref		Ref	
First-born (having only younger siblings)	**1.42**	**1.08, 1.87**	**1.58**	**1.18, 2.11**
Middle-born (having both older and younger siblings)	**1.38**	**1.11, 1.73**	**1.28**	**1.02, 1.61**
Last-born (having only older siblings)	**1.39**	**1.10, 1.76**	1.25	0.99, 1.60
Number of caries in permanent teeth in 2015	**2.06**	**1.86, 2.27**	**2.09**	**1.88, 2.32**
Number of dental clinics in a school district:				
The lowest tertile (0–3 clinics)	Ref		Ref	
The middle tertile (4–6 clinics)	0.60	0.35, 1.03	0.71	0.43, 1.18
The highest tertile (7–27 clinics)	0.69	0.39, 1.21	0.93	0.51, 1.70
Land prices in a school district (JPY/m^2^)	1.00	1.00, 1.00	1.00	1.00, 1.00
ICC (%)	22.7	22.5, 22.8	15.9	15.6, 16.1

a Adjusted for maternal age, education, paternal education, annual household income, marital status, parental psychological distress, child sex, birth order, the number of caries in permanent teeth in 2015 and number of dental clinics and land prices in a school district using a multilevel Poisson regression model with a random intercept.

Bold indicates *p* < 0.05.

Table 3 shows the association between the number of convenience stores in a school district and the number of caries in permanent teeth among fourth-grade elementary school children, stratified by household income. In children from non-low-income households, children of the school districts with the highest and middle number of convenience stores had 52% (mean ratio 0.48, 95% CI: 0.26, 0.88) and 37% (mean ratio 0.63, 95% CI: 0.37, 1.05) fewer caries in their permanent teeth, respectively, than children in the school districts with the lowest number of convenience stores. However, there was no association between the number of convenience stores in the school district and the number of caries in permanent teeth among children in low-income households. After covariate adjustment, the mean ratio of children in the school districts with the highest and middle number of convenience stores to those in the school districts with the lowest number of convenience stores were 1.09 (95% CI: 0.47, 2.56) and 1.14 (95% CI: 0.42, 3.09) respectively. We also assessed the interaction term between household income and convenience store density on caries and confirmed interaction effect (*p* < 0.1).

**Table 3:** Association between the number of convenience stores in a school district and the number of caries in permanent teeth among fourth-grade elementary school children in 2018, stratified by household income.

	Crude		Adjusted	
	Mean ratio	95%CI	Mean ratio	95%CI
Children in non-low-income households^a^ (*n* = 2,534)				
School districts with the low number of convenience stores (the lowest tertile: 0–2 stores)	Ref		Ref	
School districts with the middle number of convenience stores (the middle tertile: 3–4 stores)	0.66	0.39, 1.13	0.63	0.37, 1.05
School districts with the high number of convenience stores (the highest tertile: 5–15 stores)	**0.51**	**0.28, 0.92**	**0.48**	**0.26, 0.88**
*p* for trend	**0.023**		**0.015**	
Children in low-income households^b^ (*n* = 312)				
School districts with the low number of convenience stores (the lowest tertile: 0–2 stores)	Ref		Ref	
School districts with the middle number of convenience stores (the middle tertile: 3–4 stores)	1.09	0.44, 2.68	1.09	0.47, 2.56
School districts with the high number of convenience stores (the highest tertile: 5–15 stores)	0.89	0.32, 2.42	1.14	0.42, 3.09
*p* for trend	0.836		0.797	

a Adjusted for maternal age, education, paternal education, annual household income, marital status, parental psychological distress, child sex, birth order, the number of caries in permanent teeth in 2015 and number of dental clinics and land prices in a school district using a multilevel Poisson regression model with a random intercept.

b Adjusted for maternal age, education, paternal education, marital status, parental psychological distress, child sex, birth order, the number of caries in permanent teeth in 2015 and number of dental clinics and land prices in a school district using a multilevel Poisson regression model with a random intercept.

Bold indicates *p* < 0.05.

## Discussion

In this study, we found that children in the school districts with higher number of convenience stores had fewer caries in their permanent teeth. Further, the inverse association between the number of convenience stores and caries status in permanent teeth was observed for children from non-low-income households, but not for children from low-income households.

Previous studies have examined the association between community level exposures, such as area-level deprivation (10–12), school-based fluoride intervention programs (13), and vending machine use surveyed by questionnaire, and dental caries in children (14). The number of convenience stores is an important factor at the community level because people today frequently shop at convenience stores. In fact, previous studies showed the positive association between access to local convenience stores and diabetes and obesity (33, 34). For example, a systematic review of the association between access to convenience stores and childhood obesity reported a positive association between density of and proximity to convenience stores and obesogenic eating behaviors in children and adolescent (34). Interestingly, this systematic review also reported that an association between access to convenience store and child weight varied by region. While most studies in the US reported positive associations between access to convenience store and child weight, no such associations were found in East Asia (34). The authors argued that this may be related to the fact that both healthy and unhealthy foods may be offered in convenience stores due to regional food characteristics and regulations, and the availability of other types of food stores nearby (34). It is possible that the association between convenience stores access and dental caries may also vary by region. However, studies examining the relationship between convenience stores and dental caries other than our study are lacking. Further studies on the association between convenience stores and dental caries in other countries and regions are warranted.

Interestingly, an inverse association between the number of convenience stores and dental caries was observed in children from non-low-income households but not in children from low-income households. Previous studies conducted in Japan and the US suggested that people in low-income households are more sensitive to the price of goods than to the distance to the store, and thus tend to go to distant discount stores to purchase goods at cheaper prices instead of buying goods at neighborhood stores, especially so in large cities (30, 31). Thus, the lack of association between the number of convenience stores and dental caries among low-income households could be due to the fact people from low-income households may not purchase products that are protective against dental caries at convenience stores. Potential purchases could be fluoridated toothpaste and bottled Japanese tea, which contain a high fluoride concentration (35), especially bottled tea (36). It is true that convenience stores sell not only fluoridated toothpaste and bottled tea, but also carbonated drinks, juice, and even snacks. However, although statistics for convenience stores were not available, a statistic in Japan showed that tea beverages ranked first in terms of production share by category of soft drinks, with 5,588,000 kiloliters, followed by mineral water (4,461,300 kiloliters) in second place and carbonated drinks (3,801,200 kiloliters) in third place (37). This shows how much tea is consumed in Japan and given that Japan has good access to convenience stores, it is possible that people are buying these teas at convenience stores. Further research is needed to clarify the mechanism that explain the association between the number of convenience stores and dental caries.

This study has several limitations. First, this study was conducted in only one area of Tokyo. It is possible that the effect of convenience stores was underestimated because Tokyo has many stores other than convenience stores and has good access to public transportation, so people buy goods at discount stores other than at convenience stores. Second, in this study, the number of convenience stores in the school district was used to determine people’s access to convenience stores, but the distance from home to a convenience store was not considered. However, since the school district coincides with people’s daily living range (38), the number of convenience stores in the school district could be considered equivalent to the number of convenience stores that people have access to on a daily basis. Third, the number of convenience stores was derived from the Japanese Yellow Pages database of phone numbers, addresses, and job titles (NTT TownPage database by NTT TownPage Corporation, Tokyo, Japan) (20, 21) and not all convenience stores may have been covered. However, the total number of convenience stores in Adachi City by the three major convenience store operators is 248 (39–41), while the total number of convenience stores in Adachi City in this study is 240, which is considered to be sufficient coverage and therefore the data is considered valid. Fourth, the reliability of dentists’ diagnosis of dental caries in dental checkups by school dentists has not been evaluated; however, all dentists in Japan are required to follow national guidelines (24), and prior studies have conducted research using this information (25, 26). Fourth, we did not assess individual-level consumption behavior at convenience store, thus detailed mechanism on the inverse association remains uncovered. Further study linking purchase record at convenience store and dental caries status is warranted.

Nonetheless, our study has several implications. We found that a large number of local convenience stores are protective against dental caries. If future research can identify products sold in convenience stores that are protective against dental caries, it may be possible to reduce dental caries in the community by promoting the sale of these products not only in convenience stores, but also in other stores. Since dental caries not only leads to malnutrition and poor quality of life in children (7), but also school absenteeism and poor academic performance (8), clarification of protective products and development of such interventions are important for the well-being of children.

In conclusion, we found that children in school districts with high and middle number of convenience stores had fewer caries in their permanent teeth, respectively, than children in school districts with low number of convenience stores. Further study is needed to clarify the mechanism that explain the association between the number of convenience stores and dental caries in children.

## Data availability statement

The original contributions presented in the study are included in the article/supplementary material, further inquiries can be directed to the corresponding author.

## Ethics statement

The studies involving humans were approved by the Ethics Committee at the National Center for Child Health and Development (Study ID: 1147) and Tokyo Medical and Dental University (Study ID: M2016-284). The studies were conducted in accordance with the local legislation and institutional requirements. Written informed consent was obtained from caregivers.

## Author contributions

NN: conceptualization, data curation, methodology, formal analysis, and writing – original draft. HN: data curation and writing – review and editing. YM: methodology and writing – review and editing. SD and AI: resources and writing – review and editing. TF: supervision, conceptualization, validation, and writing – review and editing. All authors contributed to the article and approved the submitted version.
